# Dimensionality Controls Cytoskeleton Assembly and Metabolism of Fibroblast Cells in Response to Rigidity and Shape

**DOI:** 10.1371/journal.pone.0009445

**Published:** 2010-03-23

**Authors:** Mirjam Ochsner, Marcus Textor, Viola Vogel, Michael L. Smith

**Affiliations:** 1 BioInterface Group, Laboratory for Surface Science and Technology, ETH Zurich, Zurich, Switzerland; 2 Laboratory for Biologically Oriented Materials, ETH Zurich, Zurich, Switzerland; Massachusetts Institute of Technology, United States of America

## Abstract

**Background:**

Various physical parameters, including substrate rigidity, size of adhesive islands and micro-and nano-topographies, have been shown to differentially regulate cell fate in two-dimensional (2-D) cell cultures. Cells anchored in a three-dimensional (3-D) microenvironment show significantly altered phenotypes, from altered cell adhesions, to cell migration and differentiation. Yet, no systematic analysis has been performed that studied how the integrated cellular responses to the physical characteristics of the environment are regulated by dimensionality (2-D versus 3-D).

**Methodology/Principal Findings:**

Arrays of 5 or 10 µm deep microwells were fabricated in polydimethylsiloxane (PDMS). The actin cytoskeleton was compared for single primary fibroblasts adhering either to microfabricated adhesive islands (2-D) or trapped in microwells (3-D) of controlled size, shape, and wall rigidity. On rigid substrates (Young's Modulus = 1 MPa), cytoskeleton assembly within single fibroblast cells occurred in 3-D microwells of circular, rectangular, square, and triangular shapes with 2-D projected surface areas (microwell bottom surface area) and total surface areas of adhesion (microwell bottom plus wall surface area) that inhibited stress fiber assembly in 2-D. In contrast, cells did not assemble a detectable actin cytoskeleton in soft 3-D microwells (20 kPa), regardless of their shapes, but did so on flat, 2-D substrates. The dependency on environmental dimensionality was also reflected by cell viability and metabolism as probed by mitochondrial activities. Both were upregulated in 3-D cultured cells versus cells on 2-D patterns when surface area of adhesion and rigidity were held constant.

**Conclusion/Significance:**

These data indicate that cell shape and rigidity are not orthogonal parameters directing cell fate. The sensory toolbox of cells integrates mechanical (rigidity) and topographical (shape and dimensionality) information differently when cell adhesions are confined to 2-D or occur in a 3-D space.

## Introduction

The physical properties of the local cell microenvironment regulate cell behavior in concert with autocrine and paracrine soluble or matrix bound signaling molecules [1]–[5]. *In vivo*, these properties are defined by a fibrillar ECM and adjacent cells and have implications for human health and disease [6]. Our understanding of their role in regulating cell physiology resulted from technological advances which led to reductionist cell culture systems with tunable substrate stiffness, ligand density/topography, or cell adhesive area and shape in two dimensions (2-D). Most of these studies were performed on flat, 2-D culture surfaces where a number of seminal studies have shown that these properties regulate a seemingly endless variety of observable cell responses. These include spreading and migration [7]–[10], proliferation and apoptosis [11], [12], differentiation [13]–[15], and orientation of the axis of cell division [16]. Regulating these processes with engineered cell culture platforms or scaffolds might prove useful in tissue engineering or regenerative medicine applications where a specific cell phenotype needs to be stimulated or maintained [17].

Since a three-dimensional (3-D) spatial arrangement of contacts with ECM fibers or other cells is characteristic for most cells *in vivo*, the extent to which observations made in Petri dishes can be transferred to predict cell behavior in a 3-D environment has been an active area of research [17]–[22]. However, only a limited number of studies investigated the different microenvironmental parameters as a function of dimensionality. Cell culture platforms such as sandwich cultures [17], [18], gels [3], [23], [24] and cell derived 3-D matrices [20], [25]–[27] mimic the 3-D environment of a cell, but they do not allow for the control of cell shape, often have limited control over stiffness, and thus cannot be directly compared to the adhesive area of cell contact and matrix rigidity found in 2-D culture systems. Conversely, the 2-D systems, such as homogeneous or micropatterned substrates [12] or gels with tunable stiffness [14], lack a 3-D arrangement of cell contacts in the early phases of spreading. A predictive understanding of the cellular response to physical cues necessitates the development of tools that allow for independent control of surface area of adhesive contact, substrate stiffness, and dimensionality. Here, we utilized a microwell platform in polydimethylsiloxane (PDMS) that consists of an array of wells that are coated with the cell adhesion protein fibronectin (Fn) and a non-adhesive plateau that prevents cells from adhering to or crawling out of the wells [28], [29]. The microwell platform allows for control of 3-D shape, stiffness of the substrate within the range that can be replicated in PDMS (10 kPa to 1 MPa), and chemical coating specificity of the well surface. Therefore it is possible to study the contribution of these factors separately and make direct comparisons to studies on 2-D patterned surfaces.

The focus here was to determine how the surface area of adhesive contact and substrate rigidity differentially regulate actin cytoskeleton assembly in 2-D versus 3-D environments, and how this impacts cell phenotypes. Studies of cytoskeletal organization were performed because the cytoskeleton is extremely sensitive to environmental properties. The ability of a cell to migrate, apply contractile forces, and divide requires an intact actin cytoskeleton since actin filaments are force-bearing structures that link intracellular force generation to ECM attachments. Most cells probe their microenvironment as they anchor through focal adhesions, pull on their environment, and respond to the resistance that the cell senses through cytoskeletal reorganization. In return, the cell responds to the environment by altering adhesion [9], [30], [31] and cytoskeleton architecture [32], [33]. Both soft substrates and confinement of cell adhesion to small patterns in 2-D systems inhibit the formation of a filamentous actin network in many cell types [12], [34]. Importantly, the presence of an actin cytoskeleton and the ability to generate contractile forces are requisite steps in the response of cells to their environment. For example, cell shape dictates the organized structure of the cytoskeleton, which then controls the orientation of the axis of cell division [16]. Inhibition of actin cytoskeleton assembly or intracellular proteins that mediate or control cell contractility, such as nonmuscle myosin II, blocks human mesenchymal stem cells from differentiating in response to these physical cues [14]. To determine whether shape- and rigidity-dependent assembly of an actin cytoskeleton are orthogonal or dependent upon the dimensionality of cell contact with the surroundings, the actin cytoskeleton was visualized using high resolution confocal microscopy of cells on 2-D patterned surfaces and in 3-D PDMS microwells. We show that the dependency of actin cytoskeleton assembly, metabolic activity, and cell survival on the surface area of cell contact are altered in a 3-D environment.

## Results

**Figure 1:**
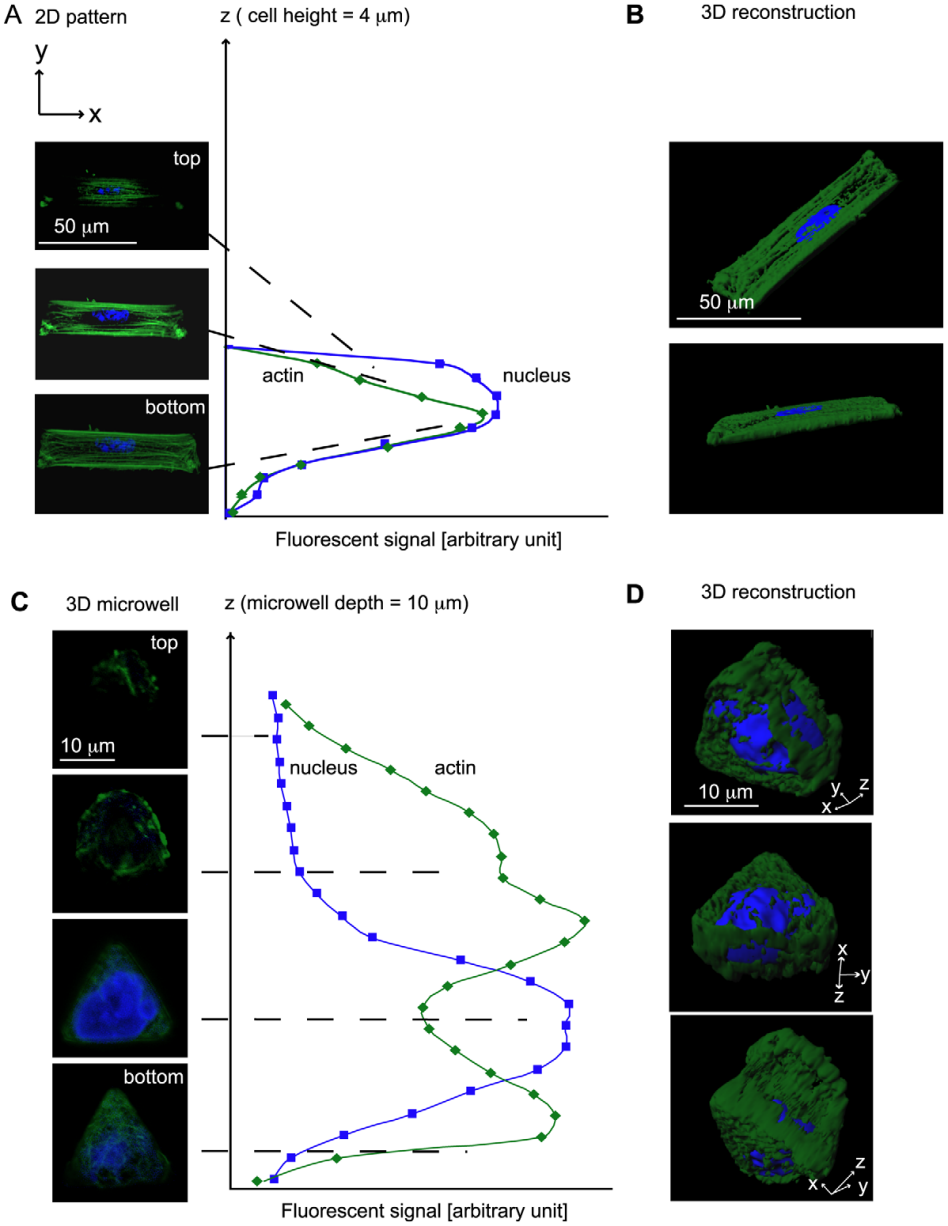
3-D organization of actin skeleton inside microwells versus 2-D surfaces. To visualize the altered actin organization on 2-D versus 3-D microwells (Young's Modulus 1 MPa), the fluorescent signals of actin (phalloidin 488) and the nucleus (ethidium homodimer) were quantified on a 2-D pattern and inside a 3-D microwell after 24 hours. A) The actin on a 2-D pattern was most abundant on the interface between the cell and the substrate. B) The reconstruction of a cell on a 2-D pattern shows a flat cell with stress fibers along the long axis but little 3-D arrangement of actin. C) The quantification of actin inside a 3-D microwell with a triangular shape, 20 µm edge length, and 10 µm depth shows actin accumulation at the interface between the cell and the bottom of the well and on top of the microwell thus covering the nucleus. The middle area of the microwell is mostly filled with the nucleus. D) The 3-D reconstruction shows shows the strongest actin staining above and below the nucleus.

To determine how a 3-D arrangement of cell adhesion to ECM molecules influences cytoskeletal organization, we investigated the spatial arrangement of actin in fibroblast cells cultured in microwells in comparison with cells on 2-D patterned surfaces of the same projected area. Two different metrics were used in this context: first, total adhesive area in the microwell refers to the surface area of the microwell floor plus the microwell walls, while 2-D projected surface area refers only to the area of the microwell floor. This was done to differentiate between cell spreading and the total area of adhesive contact. We chose to use fibroblast cells since they normally reside in a 3-D matrix *in vivo*, but are often studied on 2-D platforms *in vitro* (for example [7]). The PDMS microwells had various shapes with well volumes close to the average volume of a single cell (see Table S1 for microwell dimensions) and were coated with Fn on the bottom and walls of the microwells. The actin cytoskeleton was visualized using fluorescently labeled phalloidin. Phalloidin is unable to bind to monomeric G-actin, and hence fluorescence is only seen where filaments are present [35]. Fluorescence images were taken as high-resolution confocal z-stacks, and the intensity of the fluorescent signal of actin, an indirect indication of the presence of actin filaments, and the nucleus were quantified as function of the well depth (Fig. 1).

### Going 3-D alters actin fiber assembly in response to the surface area of adhesive contact

**Figure 2:**
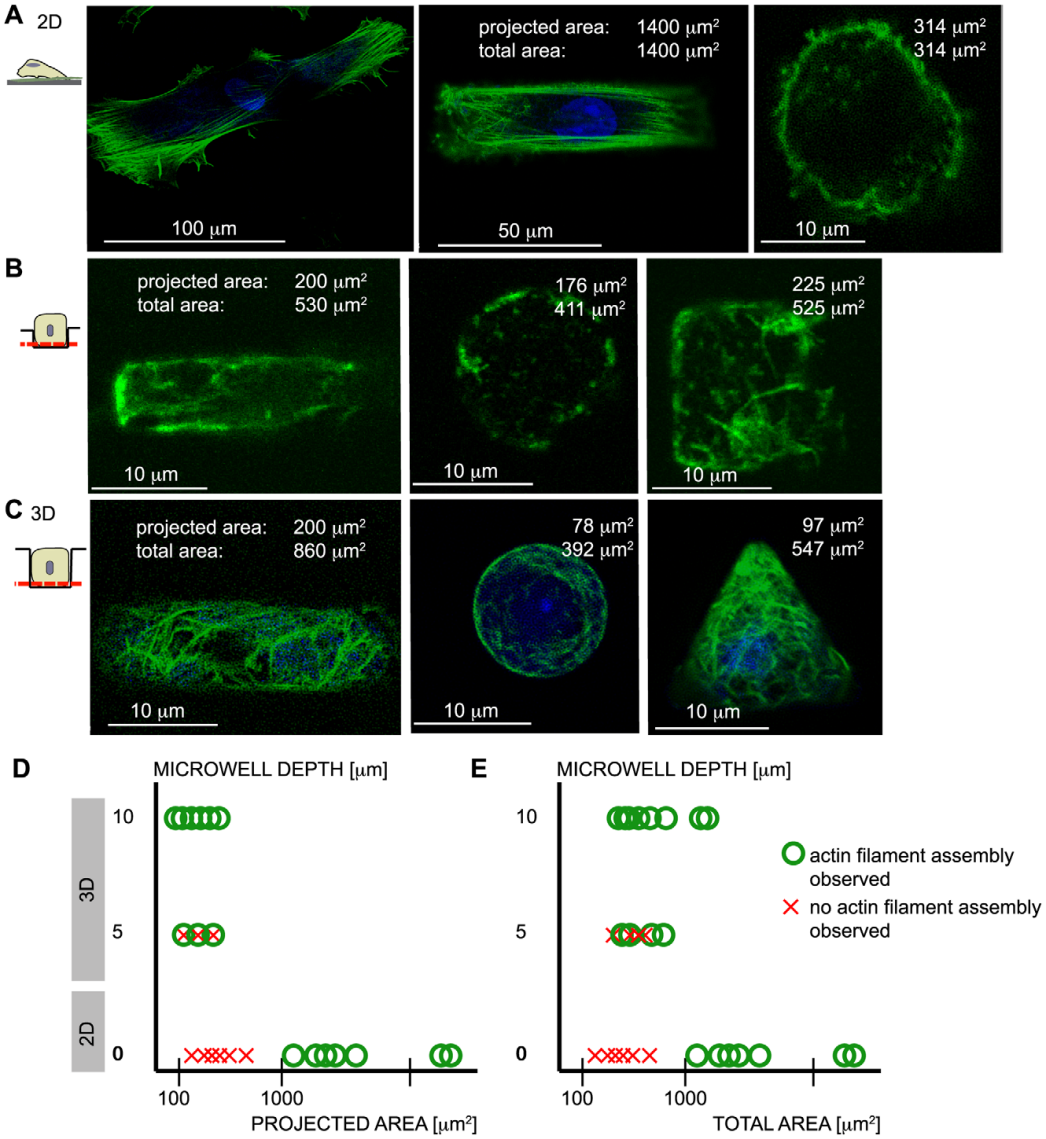
Reduction of actin filament assembly due to limited adhesive surface area is overcome by going 3-D. To test whether a 3-D arrangement of cell matrix contact altered actin cytoskeleton assembly, actin filament assembly was visualized using phalloidin Alexa 488 staining and the nucleus with ethidium homodimer on A) flat surfaces and B) in 5 µm- or C) 10 µm-deep microwells with circular, rectangular, square, and triangular shapes after 24 hours in culture. A) Cells on a 2-D surface that was homogeneously coated with Fn (no spreading limitation) or on a relatively large pattern (20 by 80 µm rectangular) formed stress fibers that were clearly visible in all imaged cells. However, cells on small patterns (12 µm circle) showed little to no actin fiber formation. B) Cells inside 5µm-deep microwells showed heterogeneous patterns of actin filament assembly where approximately half of the cells looked similar to cells on small 2-D pattern, whereas half showed actin filament assembly. C) In contrast, fibroblast cells inside 10 µm deep microwells of all shapes and 2-D projected areas assembled an actin filament network. Even if the total adhesive surface area of the floor and walls was taken into account, these cells did not assemble visible actin structures on a 2-D surface of the same projected area. D) The schematic of surface area of contact versus dimensionality shows that a cell can only assemble an actin network if the cell has a certain minimum spreading area or E) a certain total surface area. This critical range of spreading area is shifted towards smaller areas if the cell is able to interact with a 3-D surface. Shape information is provided in the text in the Results section.

On 2-D patterns on glass, phalloidin-positive actin filaments were most abundant at the interface between the single fibroblasts and the substrate (Fig. 1A). The 3-D reconstruction shows a flat cell with no prominent 3-D actin network circumscribing the nucleus (Fig. 1B). Above the cell nucleus, fewer actin fibers were visible. Confinement of cell adhesion to surface areas of ∼1000 µm^2^ and smaller led to an inhibition of actin stress fiber assembly (Fig. 2A). On bigger 2-D patterns (surface area >1000 µm^2^), fibroblasts assembled an actin network with stress fibers along the long axis of the pattern. This supports previous findings showing that limited cell spreading reduced cell contractility [36], [37]. In contrast to 2-D substrates, single fibroblasts cultured within small 3-D microwells with circular (25 cells), rectangular (20 cells), triangular (22 cells), and square shapes (23 cells) that were 10 µm deep cast in PDMS with a stiffness of 1 MPa assembled an actin filament network. Note that all data herein represents an ensemble of single cell measurements using all four microwell shapes, and each trend shown was found to be independent of the tested well shapes. Actin filaments in 3-D wells appeared as an entangled network with little or no straight stress fibers visible. Phalloidin-positive actin filaments were often found above the nucleus, in contrast to cells on 2-D patterns. Integrated fluorescence intensities as a function of z-position (Fig. 1C) and a 3-D reconstruction (Fig. 1D) confirmed this trend.

Fibroblasts were next cultured within 3-D microwells and on 2-D patterned surfaces with similar surface areas of adhesive contact in order to clarify whether this actin filament assembly was a function of contact dimensionality. Cells in microwells rarely had a flat upper surface. However, the bottom plane of the microwell always resulted in a flat cell contact surface, and confocal images from this plane were used for figure presentation since the actin structures were parallel to and contained within the imaging plane (i.e. parallel to the microwell bottom; Fig. 2C). Every cell cultured within 10 by 10 µm square (100 µm^2^ of 2-D projected area: microwell bottom, and 500 µm^2^ of total adhesive contact area: sum of wall and bottom areas) or 10 µm diameter circular microwells that were 10 µm deep (78 µm^2^ of 2-D projected area and 392 µm^2^ of total adhesive contact area) stained positive for phalloidin (Fig. 2D, E). This total surface area of contact is well below the contact area of ∼1000 µm^2^, which is the minimum adhesive pattern area in 2-D that allows for actin assembly (Fig. 2E). Since we were unable to quantify the amount of actin filament assembly inside microwells using fluorescence, a library of confocal images of the bottom plane of different single cells inside microwells is provided in order to demonstrate the reproducibility of actin assembly within 3-D microwells (Fig. S1). Interestingly, we did not find any correlation between actin fiber formation and microwell shape.

In order to exclude the possibility that Fn density or anchorage on patterned glass surfaces was insufficient to promote actin polymerization that was permitted in PDMS microwells, we next asked whether actin assembly occurs when cells are cultured in 5 µm deep wells (Figs. 2B, S2). At this intermediate depth, some cells were able to assemble actin filaments in very confined microwells similar to the 10 µm deep wells, whereas others showed no actin network, similar to a small 2-D pattern. This behavior was not microwell shape dependent, as 10 of 19 cells and 8 of 17 cells in square and circular microwells, respectively, assembled actin structures. The range of 2-D projected area was the same for both shallow and deep microwells (78–330 µm^2^), but the total adhesive surface area was between 300–690 µm^2^ for shallow wells relative to 314–942 µm^2^ for deep microwells. From this data, we conclude that cell confinement and substrate rigidity, but not Fn density or anchorage, controls actin polymerization and that the decision of a fibroblast to assemble an actin cytoskeleton is an all or none response, with some cells not responding to dimensionality at these intermediate well heights (Fig. 2D, E). We cannot exclude the possibility that cell-to-cell variability in the formation of actin fibers in shallow wells reflects potential differences in cell volume, although as it is not possible to estimate cell volumes from our data.

### Going 3-D also alters actin cytoskeleton assembly in response to substrate stiffness

**Figure 3:**
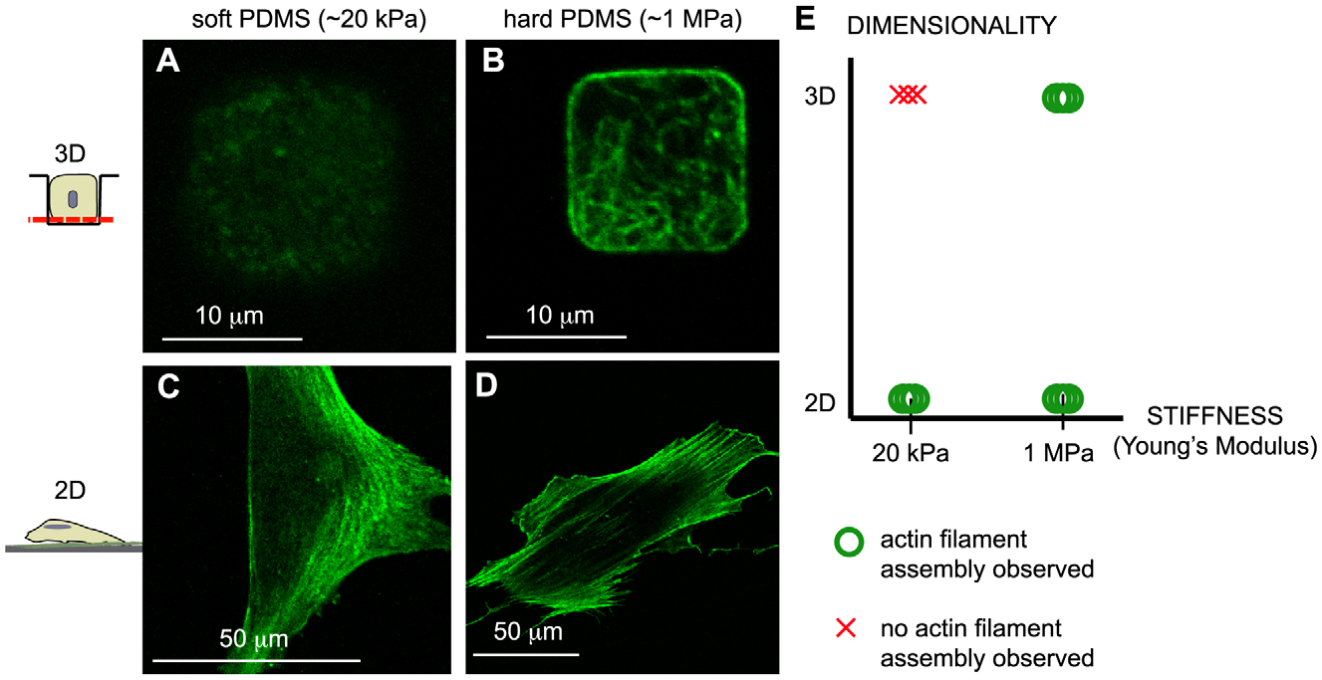
Dimensionality alters the rigidity below which assembly of the actin cytoskeleton is diminished. In order to test whether substrate stiffness impacted the stimulation of actin filament assembly in a 3-D environment, fibroblast cells were cultured for 24 hours on homogeneously Fn-coated 2-D PDMS surfaces and in 3-D PDMS microwells with a Young's Modulus of 20 kPa (A and C) or 1 MPa (B and D), and actin fibrils were visualized with phalloidin Alexa 488. A) Actin filament assembly was inhibited inside soft 3-D microwells (15 by 15 µm square, 10 µm deep shown), B) but cells formed actin stress fibers inside hard 3-D microwells (15 by 15 µm square, 10 µm deep shown). C) In 2-D, cells formed actin stress fibers on soft as well as D) on hard flat substrates. E) The schematic of stiffness versus dimensionality shows that a cell can only build up an actin network if the substrate has a certain minimum stiffness. This critical range of stiffness is shifted towards higher stiffness if the cell is able to interact with a 3-D surface. Microwell shape information is provided in the text in the Results section.

Sensory systems that probe different environmental properties are hypothesized to be intimately linked. For example, substrate stiffness affects the extent of cell spreading and hence cell shape, indicating that cell responsiveness to substrate stiffness in 2-D could be due to alterations in cell shape. To address whether dimensionality alters cell sensitivity to substrate rigidity, cells were cultured on flat substrates and inside circular (25 cells), square (22 cells), and triangular (14 cells) microwells that were cast in either hard (∼1 MPa) or soft PDMS (∼20 kPa). Here, the microwells in both soft and hard PDMS were limited to 2-D projected surface areas between 78 and 330 µm^2^ (total surface areas of 314–942 µm^2^) so that substrate rigidity was tested for two populations of single cells in microwells that had similar cell adhesion surface areas, but different rigidities. On flat 2-D substrates, cells formed phalloidin-positive actin structures for the two cases of high and low substrate stiffness (Fig. 3D, C). The substrate stiffness below which fibroblasts on 2-D surfaces were incapable of making an actin cytoskeleton was below 20 kPa, the softest substrate testable here with PDMS. Importantly, cell survival was similar for hard and soft PDMS (Fig. S3), indicating that there was no toxic effect of the soft PDMS on cells.

In 3-D microwells of a projected area between 78 and 330 µm^2^ (total surface areas of 314–942 µm^2^), the cells were able to assemble an actin network in contact with a hard substrate (∼1 MPa; Fig. 3B). Remarkably, in softer microwells, cytoskeleton assembly was diminished for the same range of microwell sizes (∼20 kPa; Fig. 3A, S4). Although the same microscopy settings were used for both hard and soft microwells, no actin filaments were detectable in any of the cells in soft microwells (Fig. S5). This was also not a result of microwell fabrication since cells cultured on microwell chips that were not passivated on the plateau surface to prevent cell adhesion assembled filamentous actin (data not shown). Thus, actin filament assembly was suppressed only in the soft microwells but not on the soft, 2-D surfaces. Again, this finding was not microwell shape dependent, since none of the cells in any of the microwells formed visible actin structures. A cell experienced the same stiffness on soft 2-D substrates and inside soft 3-D microwells, but 3-D stimulation of actin filament assembly required a threshold substrate stiffness that was dramatically higher than that required for actin filament assembly for cells on 2-D culture surfaces (Fig. 3E). The substrate rigidity above which actin cytoskeleton assembly occurred was shifted towards harder substrates in a 3-D environment compared to 2-D. Thus, mechanosensing properties and actin filament assembly are not solely dependent upon substrate stiffness, but these sensory systems also detect dimensionality and integrate this information in the process of defining a cellular response.

### Fibronectin assembly and rearrangement

**Figure 4:**
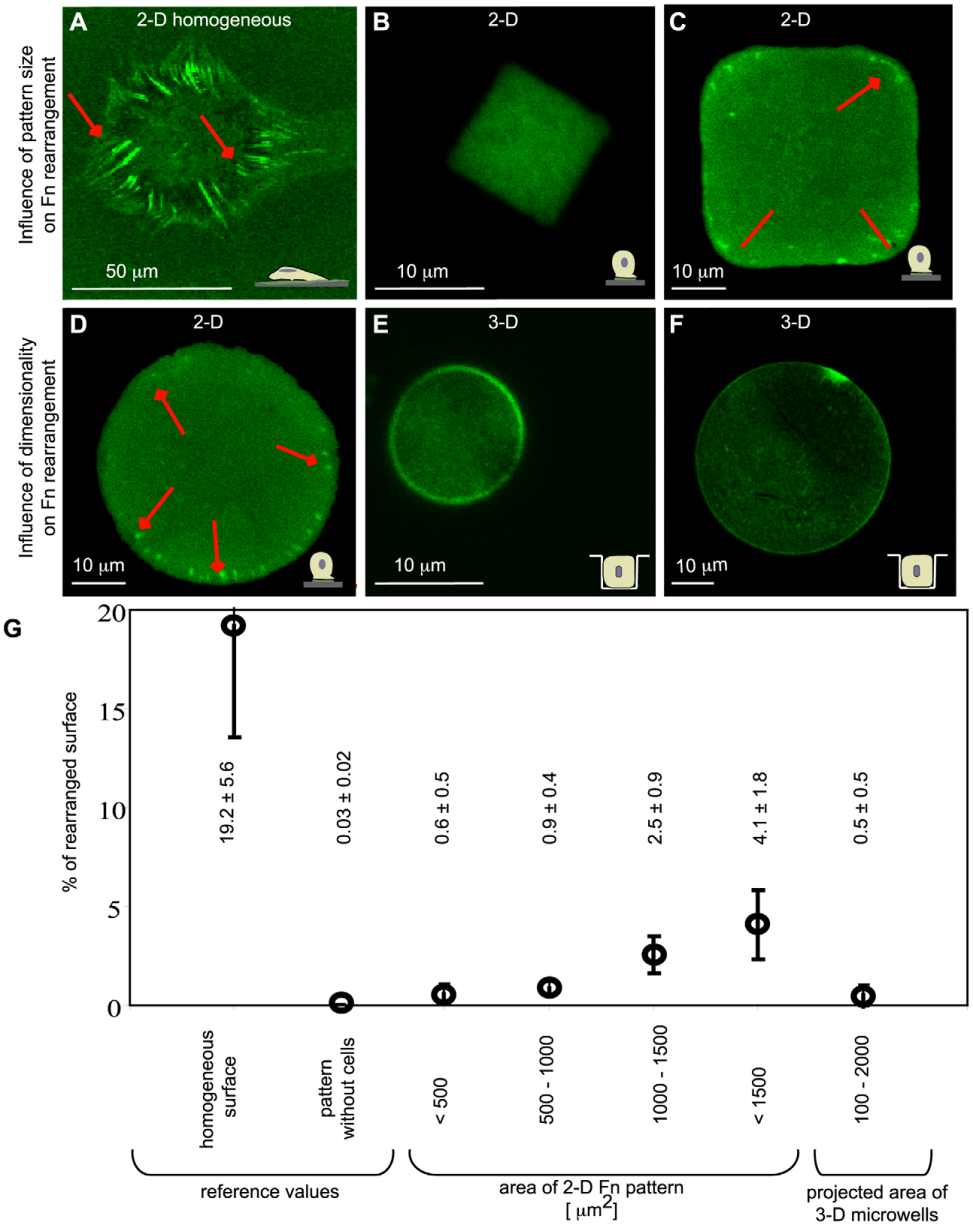
The dependence of surface adsorbed Fn rearrangement on spreading area and dimensionality. The influence of spreading area and dimensionality on the rearrangement of surface adsorbed Fn (Fn, fluorescently labeled with Alexa 488) was investigated on homogeneous and patterned 2-D and on 3-D microwell culture substrates after 90 minutes in cell culture. A) Cells on homogeneously coated 2-D surfaces rearranged the majority of surface adsorbed Fn. The bright green areas indicate an accumulation of Fn, while the darker areas indicate removed Fn. B) Cells with limited spreading area on small Fn 2-D pattern were not able to rearrange the Fn matrix. C) With increasing pattern size/spreading area, cells increased the percentage of surface adsorbed Fn that was rearranged. Square-shaped (2500 µm^2^ of total surface area) and D) circular patterns (1900 µm^2^ of total surface area) demonstrate that cells rearrange Fn from the edges. Cells inside 3-D microwells were rarely able to rearrange Fn on the bottom of the well. E) Culture of cells in small microwells (<500 µm^2^ projected area) hindered the Fn rearrangement similar to small 2-D pattern, F) but even cells in bigger microwells (>1500 µm^2^ projected area) did not allow for Fn rearrangement. G) Matlab calculations allowed for quantification of the percentage of Fn surface area rearranged by the cell. Details of the analysis are provided in Fig. S8.

Next, we determined whether confinement within 3-D microwells impacts cell contractility. Since it was not technically possible to make traction force measurements within 3-D microwells, an indirect approach was instead utilized. Fn is often used as an adhesive coating on biomaterial surfaces, and cells rearrange this surface adsorbed matrix protein into Fn fibrils in a process that requires generation of cell contractile forces that can overcome the adhesion strength of Fn to the underlying surface [38]. Cell-dependent rearrangement of fluorescently labeled, surface adsorbed Fn was imaged after 90 minutes in cell culture since the early time point minimized the contribution of cell-derived Fn to fiber formation. First, 2-D Fn patterns on glass with protein islands and a cell resistant background were produced by the MAPL technique [39] in order to determine whether the surface area of cell adhesion, which was shown to influence cell contractility [40], also impacts rearrangement of surface adsorbed Fn into fibrils. Quantitative analysis of the rearranged surface area on 2-D patterns showed that cell confinement progressively limited Fn rearrangement of surface-bound Fn with decreasing pattern size (Fig. 4A–D, G). The percentage of Fn rearrangement increased with increasing pattern size from 0.6±0.5% rearranged surface area on <500 µm^2^ patterns to 4.1±1.8% on larger, >1500 µm^2^ patterns.

Fn rearrangement was also investigated in the hard 3-D microwells (1 MPa). Within very small 3-D microwells (78–200 µm^2^ projected surface area/314–765 µm^2^ total surface area), Fn rearrangement into fibrils on the bottom surface of the well was inhibited (Fig. 4E). In bigger microwells (1100–1600 µm^2^ projected surface area/2426–3200 µm^2^ total surface area), no Fn rearrangement occurred (Fig. 4F), which was in contrast to the 2-D patterns of the same size where the fibroblasts were able to rearrange Fn. However, we cannot exclude the possibility that Fn rearrangement occurred on the vertical walls since these surfaces were perpendicular to the confocal imaging plane and therefore difficult to image. Control experiments showed that Fn rearrangement also did not occur after 24 hours of culture within microwells (Fig. S6). Furthermore, fluorescent immunohistochemical labeling of human Fn did not reveal any Fn fibers after 24 hours culture, indicating that cell-derived Fn was not used to produce Fn matrix fibers in the absence of adsorbed Fn rearrangement.

### Cell metabolism is dependent on spreading area and upregulated by going 3-D

**Figure 5:**
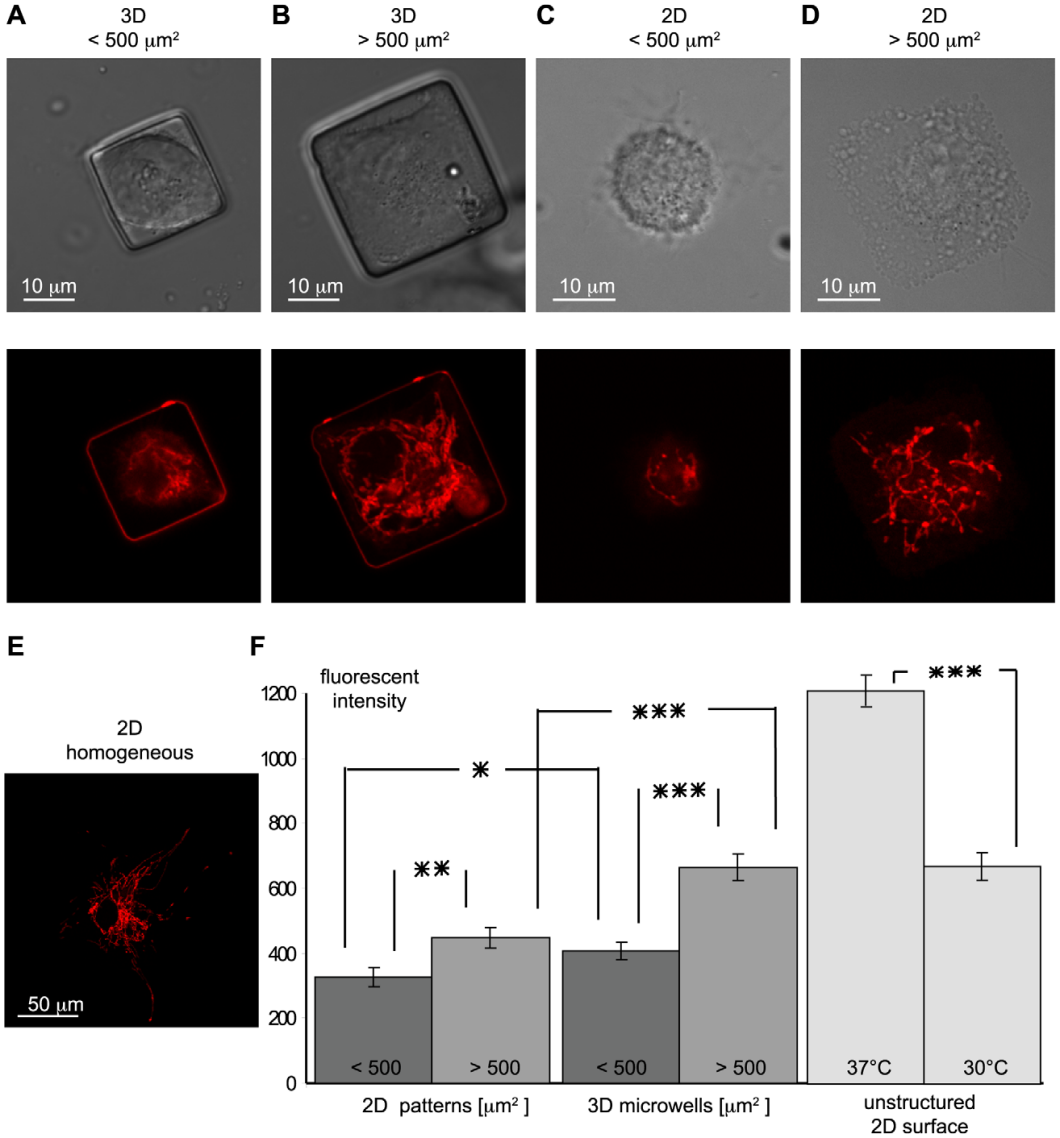
The dependence of cell metabolism on dimensionality and spreading area. To investigate the influence of spreading area and dimensionality on cell metabolism, cells were cultured for 24 hours A) inside hard (Young's Modulus 1 MPa), small 3-D microwells with circular (10 microwells) and square (23 microwells and 2-D projected surface area <500 µm^2^, B) big microwells with circular (10 microwells) and square (15 microwells) and 2-D projected surface area >500 µm^2^, and C) on small and D) big 2-D patterns and stained for mitochondrial membrane potential (TMRE). E) As a reference, cells were cultured on homogeneously coated surfaces at 30°C and 37°C. F) The quantitative fluorescent signals obtained from adding z-stacks show the difference in metabolic activity depending on dimensionality and spreading area. Average fluorescent intensity and the standard error of the mean are plotted (*<0.05, **<0.01, ***<0.001).

The metabolism of a cell is dependent on mitochondrial activity. To investigate the dependence of the metabolism of cells on contact area and dimensionality, the mitochondrial membrane potential was determined by staining the cells with TMRE (Fig. 5A–E). The fluorescent intensity, a measure of mitochondrial membrane potential, was integrated in each confocal slice in order to compare the metabolic activity of single cells in different culture systems (Fig. 5F). As a control, mitochondrial membrane potential was imaged and quantified in cells at 37°C and 30°C. The more intense signal for cells at 37°C, in comparison to cells at 30°C, indicated the greater metabolic activity of cells at 37°C, as expected (p<0.001). This indicated that this method could be used to quantify the fluorescent signal and thereby infer the metabolic activity within cells.

Finally, we asked whether environmental properties that were shown to influence actin cytoskeleton assembly affected the metabolic activity of cells. Inhibition of cell spreading by spatial confinement is shown here to decrease the metabolic activity of cells: On 2-D patterns and inside 3-D microwells, the metabolic activity was elevated for cells with a total surface area of cell contact greater than 500 µm^2^, when compared to smaller patterns. The difference between the metabolic activity on small and big 2-D patterns (p<0.01) and 3-D microwells (p<0.001) was statistically significant. Dimensionality itself also influenced the metabolic activity. There was a small, but statistically significant increase in activity in the small 3-D microwells relative to 2-D controls (p<0.05). The difference in metabolic activity between cells on big (>500 µm^2^) 2-D patterns and within big 3-D microwells was even more pronounced (p<0.001). In general, we conclude that the metabolic activity of a cell was affected by the dimensionality of a cell and was increased in a 3-D environment.

### For a given surface area of cell adhesion, cell viability is increased by going 3-D

As reported before for 2-D islands, inhibition of cell spreading by confining cell adhesion area reduced cell viability [12]. In agreement with previous studies, we find that cell viability on small 2-D patterns was decreased, with only 36% of the cells being viable after 24 hours [28], similar to previous reports. On larger 2-D patterns, cell survival was similar to cells on a homogeneous, Fn-coated surface [12]. In contrast to 2-D, cells in microwells did not show a decreased viability due to limited contact area [28]. However, it was not possible to directly compare 3D microwells with very confined 2D data points since 100 µm^2^ of total microwell surface area (microwell floor plus walls) could only be produced in microwells that had volumes much less than the volume of cells.

## Discussion

By using microwell arrays, we could show that various cell functions are significantly altered whether cells are limited to contacting a 2-D surface, or whether additional adhesions can be formed with perpendicular walls. The topographical pattern of cell anchorage regulates the assembly of actin fibers (Fig. 2), mitochondrial activity (Fig. 5), and even cell viability in response to the surface area of cell adhesion and substrate rigidity (Fig. 3). Actin cytoskeleton assembly was studied due to its pervasive demonstration as an important coupling mechanism between external stimuli and downstream cellular responses [7], [34]. This study showed that substrate dimensionality, substrate rigidity, and cell adhesion area are not orthogonal stimuli that regulate actin cytoskeleton assembly independent of one another, but rather that fibroblast cells integrate all three stimuli in a manner that depends on the anchorage geometry (2-D versus 3-D). The data are summarized in Table 1 and support recent observations that experiments using 2-D cell culture platforms may not accurately predict the behavior of cells in their native, 3-D context [19].

**Table 1:** Influence of the variation of surface area and substrate stiffness on actin fiber formation.

			2D adhesive islands	3D microwells
**surface area (µm^2^)**	projected (microwell bottom)	78–330	*no actin fiber formation*	**actin fiber formation**
		400/600	*no actin fiber formation*	**actin fiber formation**
		900	**actin fiber formation**	**actin fiber formation**
	total (microwell bottom plus sidewalls)	314–764	*no actin fiber formation*	**actin fiber formation**
		856–1054	**actin fiber formation**	**actin fiber formation**
**stiffness (kPa)**	soft PDMS	20	**actin fiber formation**	*no actin fiber formation*
	hard PDMS	1000	**actin fiber formation**	**actin fiber formation**

### Cell shape/cell function relationships deduced from 2D surfaces might not readily be applicable for cells in 3-D environments

Cell shape plays an essential role in regulating cell behavior. For example, the control of cell adhesion area on 2-D patterned surfaces regulates cell cycle progression and the commitment of stem cells to different lineages through control of endogenous RhoA activity and cytoskeletal tension [13], [41]. It is also well established that cell shape on 2-D patterns regulates cytoskeletal structure [41] and thereby cell contractility [40] since the actin cytoskeleton is a force-bearing network that is necessary for application of cell contractile forces to the surroundings [42]. Previous studies analyzing cell functions in response to both cell shape and surface area of adhesive contact were, to the best of our knowledge, exclusively limited to engineered 2-D surfaces. Here, we find that a more confined cell, i.e. a smaller surface area of cell contact to cell volume ratio, inhibits actin assembly if the cell is restricted to form contacts exclusively to a 2-D substrate, but not if additional contacts can be formed in a 3-D context (Fig. 2). This is significant, since it implies that cell shape/cell function relationships that are deduced from using engineered 2-D surfaces might not readily be applicable to cells in 3-D environments.

The formation of a 3-D actin network by cells confined to microwells suggests that cells are capable of generating contractile forces, yet single cells were not able to rearrange adsorbed Fn into fibrils when cultured in microwells or to assemble a fibrillar Fn matrix. Cells can generate forces with vectorial components both parallel and perpendicular to a surface, and we cannot exclude the possibility that confinement within 3-D microwells leads to force applications with larger perpendicular components with respect to the walls. Future studies will explore whether the reduced force vector in the membrane plane might lead to an inhibition of Fn rearrangement and ECM assembly.

### 3-D alters rigidity dependent control of actin cytoskeleton assembly

The stiffness of the substrate also fundamentally impacts cell behavior in both 2-D and 3-D. Substrate stiffness has been shown to regulate actin cytoskeleton assembly in both 3-D gels and on 2-D substrates [24], [34], [43], [44], but no study has independently varied cell spreading area, dimensionality, and substrate stiffness. In order to inhibit actin cytoskeleton assembly in 2-D, most cell types must be cultured on very soft substrates, such as polyacrylamide gels, that reach stiffness values (less than 1 kPa) that are less than those that can be achieved with PDMS [34]. It has been shown that actin stress fiber formation and cell spreading are absent for many cell types on very soft substrates, although this behavior is cell-type specific [8], [45]. However, in 3-D, the stiffness below which cells do not assemble stress fibers was shown here to be much higher, and hence PDMS was well suited for this study since the soft PDMS (20 kPa) did stimulate actin filament assembly on flat, 2-D surfaces but not in 3-D microwells (Fig. 3). An angular component to rigidity sensing might play a so far undefined role in the sensation of 3-D environments.

### Metabolic activity and cell survival

Since numerous studies illustrated that the sensitivity of cells to substrate rigidity was correlated with a down-regulated cell contractility [7] and RhoA activity [37], we asked whether dimensionality for a given surface area of cell contact has an effect on the total metabolic activity. The metabolism of cells in 3-D microwells was upregulated compared to cells with the same substrate stiffness and extent of cell spreading on 2-D surfaces (Fig. 5), suggesting that altered cytoskeleton assembly may hint at a broader responsiveness of cells to 3-D adhesion.

In conclusion, the importance of understanding the intimate relationship between local microenvironmental properties and cell function has recently been illustrated with a number of key studies. For example, matrix stiffness has been proposed to regulate the malignant phenotype of cancer cells [46], the geometric arrangement of cell contacts was shown to alter the response of skin cells to cytotoxic agents [47], and the susceptibility of cells to non-viral gene delivery depends upon the stiffness of 2-D culture substrates [48]. A thorough understanding of the cell sensory toolbox might allow the development of engineered tissues or the maintenance of cell phenotypes, such as hepatocytes, that are important in drug toxicity screening. The data presented here confirm, along with previous studies, that 3-D contact is part of this toolbox, and specifically this data indicates that these stimuli are integrated by the cell and do not function as independent cues. The different microenvironmental properties, such as rigidity, cell spreading area, and geometric arrangement of cell contact, are not orthogonal to one another. Rather, cell viability does not correlate with any of the parameters in this study. A cell viability above 89% was seen in the 3D context for all sizes and geometries tested, but was shown to when cells were confined to spread on 2-D islands of only 100 um^2^ [12]. In addition, cell metabolic activity did not appear to correlate with formation of an actin cytoskeleton since cells in confined, stiff 3-D microwells had reduced metabolic activity compared to cells in spread, stiff 3-D microwells although both groups of cells formed an actin cytoskeleton. Hence, the interrelationship between cell viability, metabolic activity, cell shape/dimensionality, and the formation of an actin cytoskeleton is a complex function of all three variables. These parameters should be varied independently if we are to fully map the cell sensory toolbox, and we speculate that cell responsiveness to other stimuli, for example soluble signaling molecules or gradients in physical properties such as concentration or stiffness, might also be affected by 3-D versus 2-D environments. Future studies in this area should consider that 2-D *in vitro* models might not accurately predict 3-D cell behavior.

## Materials and Methods

### Fibronectin isolation and fluorescent labeling

Human plasma fibronectin (Fn) was isolated from fresh human plasma (Swiss Red Cross) using gelatin-sepharose chromatography and established methods [49], as described in [28].

### PLL-*g*-PEG

Poly(L-lysine)-*graft*-poly(ethylene glycol) (PLL-*g*-PEG; SuSoS, Switzerland) is a graft copolymer whose polycationic backbone adsorbs electrostatically on negatively charged surfaces rendering it resistant to non-specific protein adsorption [50]. The architecture of the PLL-*g*-PEG used in this study was PLL(20 kDa)-*g*-[3.4]-PEG(2 kDa) with g denoting the ratio of lysine monomers per PEG side chain.

### 2-D chemically patterned glass surfaces

2-D micropatterned glass substrates for the study of actin organization and Fn rearrangement where produced by the Molecular Assembly Patterning by Lift-off technique (MAPL) similar to Falconnet et al. [38]. Briefly, the pattern was first transferred into a Shipley 1818 photoresist (Microresist, UK), which was spin-coated on glass slides. Subsequently, the prepatterned surfaces were modified chemically to transfer the resist pattern into a biological contrast. To this end, PLL-g-PEG (0.1 mg/ml in PBS, 1 hour) was adsorbed on the surface, adsorbing on both substrate surface and photoresist. In a second step, the photoresist (including adsorbed PLL-g-PEG on top of it) was removed with methylpyrrolidone (NMP; Fluka, Switzerland), followed by a backfill of the bare glass regions with Fn (Fn, 25 µg/ml in PBS, 1 hour). The result is a molecularly flat, 2-D patterned surface with Fn islands in a PLL-g-PEG background that resists protein adsorption and cell attachment.

### 2-D homogenous and 3-D well substrates in PDMS of varying stiffness

For the production of the microwell arrays and the flat PDMS substrates, polydimethylsiloxane, PDMS (Sylgard 184, Dow Corning, US), a crosslinked siloxane polymer, was used. Its stiffness can be controlled by varying the crosslinking density. Mixtures of 1∶10 (weight ratio, w/w curing agent to prepolymer) for hard substrates (Young's modulus ∼1 MPa) and 1∶40 for soft substrates (∼20 kPa) were produced (for more details on the fabrication and Young's modulus measurement: see [28]). The PDMS was cured at 80°C for 4 hours.

### Fabrication of microwell substrates in PDMS

Arrays of microwells with different geometries (circles, squares, triangles, rectangles, spindles, etc.), different lateral dimensions (78 µm^2^ to 900 µm^2^ projected area) and a depth of 10 or 5 µm were first produced in silicon using standard photolithography with the negative photoresist SU8 (MicroChem, US) and replicated into a PDMS master (negative structure). An overview of geometries and patterns used is provided in Table S1. To achieve thin (compatible with inverted stage microscopy), microstructured PDMS films with positive structure, the structure was replicated a second time. The replication and surface modification was performed as described in (20). Briefly, after fluorosilanization of the PDMS master, the second replication resulted in the final microwell structure in PDMS with varying stiffness, depending on the chosen crosslinking density (see above). After air plasma treatment to convert the PDMS surface to a hydrophilic, SiO_2_-like thin layer, the plateau areas were passivated with PLL-*g*-PEG using an inverted microcontact printing technique and the wells were finally backfilled with Fn, resulting in the same biochemical contrast as for the 2-D patterned surfaces (see Fig. S7).

### Cell culture

Primary human foreskin fibroblasts (HFF; ATCC, US) were maintained for less than 8 passages in Fibroblast Growth Medium plus Supplement (10% serum; PromoCell, Germany). Fn coated microwells were seeded with 5*10^4^ cells/cm^2^. Cells were allowed to adhere within the microwells for 15 minutes, after which the unbound cells on the non-adhesive background were removed by gentle pipetting. For all conditions, cells were seeded into circular, rectangular, triangular and square-shaped wells. On 2-D patterned substrates, cell seeding density was 10^4^ cells/cm^2^ and the cells were allowed to adhere for 1 hour before rinsing. For actin and nuclei visualization, cells were cultured for 24 hours in 10% serum prior to cell staining and imaging. For Fn rearrangement, cells were cultured for 90 minutes in 10% serum media.

### Visualization of actin and nuclei

Images of the cells were acquired with an Olympus FV1000 confocal microscope with an oil immersion 1.35NA 60× objective. For actin stress fiber imaging, cells were permeabilized with 0.1% Triton X-100 plus 1.5% formalin in PBS for five minutes and rinsed with PBS. Afterwards, they were fixed in 3% formalin in PBS for 10 minutes. After rinsing with PBS, samples were blocked in 4% bovine serum albumin (Sigma-Aldrich, Switzerland) in PBS for 1 hour. Finally, samples were rinsed carefully and simultaneously incubated with Alexa Fluor 488 Phalloidin (1∶400 dilution; Molecular Probes, Switzerland) and ethidium homodimer-1 (1.5 µM; Molecular Probes, Switzerland) for 20 minutes. Samples were rinsed thoroughly prior to imaging.

### Fibronectin rearrangement

To assess the rearrangement of surface adsorbed Fn within microwells and on 2-D surfaces, samples were coated with Fn labeled with Alexa 488 (Molecular Probes, Switzerland) prior to cell seeding. After 90 minutes, samples were fixed and blocked as described above. Because we analyzed the rearranged Fn after 90 minutes, only surface adsorbed Fn was fluorescently labeled and imaged To quantify the surface area where Fn rearrangement occurred, a Matlab analysis was performed (Fig. S8). Briefly, the fluorescent signal of Fn labeled with Alexa 488, which was adsorbed on the surface, was measured without cells. Hence the background intensity distribution of the homogeneous coated Fn was obtained. The highest and lowest intensity values were further used as upper and lower intensity threshold values. The intensity of the Fn area below a cell was measured with these threshold values, determining the percentage of surface below (removal of Fn) and above (accumulation of Fn) the threshold. The sum of this two values resulted in the total percentage of the area where Fn rearrangement occurred.

### Mitochondrial membrane potential

To determine the mitochondrial membrane potential and therefore the metabolic activity of cells cultured on 2-D surfaces or in microwells, after 24 hours in culture HFF cells were stained with tetramethylrhodamine ethyl ester (TMRE; Molecular Probes, Switzerland). First, it was investigated whether differences in fluorescent intensity could be related to different metabolic activities. Therefore, the cells were kept for 30 min at either 30°C or 37°C in 10% serum media and 5% CO_2_ and imaged. The imaging of cells cultured on 2-D patterns and inside microwells was always performed at 37°C. To analyze the images, Matlab was used and the average fluorescent intensity of a cell determined. In order to quantify the fluorescent intensity of TMRE, three confocal image slices from the bottom, middle, and top of each cell were acquired. Next, the background value was determined and subtracted from each image. Finally, the average of all pixels above a threshold value of three times the background standard deviation was calculated for the three slices for each cell for Figure 5. This background subtraction and thresholding ensured zero intensity pixels were excluded so that the average values included only TMRE-positive pixels.

## Supporting Information

Figure S1 Library of different microwell shapes and actin formation in 10 µm deep, hard wells. Actin filament assembly of primary fibroblast (HFF) cells was visualized using phalloidin Alexa 488 (green), the nucleus with ethidium-homodimer (blue). This figure shows the reproducibility of the actin formation inside small, 10 µm deep microwells. This figure provides a library of different cells inside microwells (circles, squares, triangles, rectangles).
(5.14 MB TIF)

Figure S2 Library of actin skeleton of cells inside shallow microwells (5 µm). Actin filament assembly of primary fibroblast (HFF) single cells was visualized using phalloidin Alexa 488 (green), the nucleus with ethidium-homodimer (blue). It shows the actin formation inside small, 5 µm deep microwells. This figure provides a library of cells inside microwells of different shapes (rectangle, circles, squares).
(4.62 MB TIF)

Figure S3 Cell survival of HUVECs in dependence of dimensionality and PDMS stiffness. Human umbilical vein endothelial cells (HUVECs) were cultivated on 2-D and inside 3-D microwells on both soft (20 kPa) and hard (1 MPa) PDMS. This shows that the stiffness of the PDMS does not reduce the cell survival.
(0.14 MB TIF)

Figure S4 Library of actin skeleton of cells inside 10 µm deep, soft (20 kPa Young's Modulus) microwells. Actin filament assembly of primary fibroblast (HFF) cells was visualized using phalloidin Alexa 488 (green). The figure shows the reproducibility of the A) hindrance of actin filament assembly inside soft, 10 µm deep (∼20 kPa) microwells. This figure provides a library of different cells inside microwells (circles, squares, triangles). B) Cells on a flat 2-D, homogeneously coated substrate of the same stiffness showed clear actin filament formation.
(3.24 MB TIF)

Figure S5 Stack of actin skeleton of cells inside a soft microwells (20 kPa). Actin filament assembly of primary fibroblast (HFF) single cells was visualized using phalloidin Alexa 488 (green). It shows the absense of actin fibers inside a soft (20 kPa) microwell in contrast to hard microwells. This figure demonstrates the lack of actin fibers on the different levels inside the cells.
(0.42 MB TIF)

Figure S6 Rearrangement of surface adsorbed Fn in 3-D after 24 hours. The influence of time on surface adsorbed Fn rearrangement (Fn, fluorescently labeled with Alexa 488) was investigated on 3-D microwell culture substrates after 24 hours in cell culture. After 24 there was no rearrangement of surface adsorbed Fn observable which corresponded to substrates after 90 minutes.
(0.29 MB TIF)

Figure S7 Production by replication and surface modification of the microwell substrates. A) First, the microwell structure of the silicon wafer (positive structure) was replicated into PDMS with the latter serving as master (negative structure) for the subsequent replication step. After fluorosilanization of the PDMS master, the microwell structure was replicated into a thin PDMS supported on a glass cover slip (total thickness<170 µm, compatible with inverted-stage high-resolution fluorescence microscopy). The microwell plateau surface was next passivated by inverted micro-contact printing of PLL-g-PEG using a polyacrylamide stamp. The stamp was placed upside down on the structured surface, and a 5 g weight was applied. After passivation of the plateau, the sample was finally exposed to a solution containing fibronectin (Fn) for coating of the wells; after rinsing only the microwell surfaces (bottom and walls) were coated with the cell-adhesive protein. B) Cartoon of microwells with well volumes that can be tailored to the volume of a single cell resulting in single cells in wells that interact with ECM molecule inside the well while the plateau areas resist protein adsorption and cell adhesion. C) Scanning electron microscopy images of the microwell surface show wells with different shapes and a zoom into a single triangular well. D) The scanning laser confocal microscope image shows the side view of a microwell selectively coated with fluorescently labeled Fn (green). E) A fibroblast cell adhering inside a microwell was visualized with actin (Phalloidin 488, green) and nuclei staining (ethidium homodimer, blue).
(0.70 MB TIF)

Figure S8 Analysis of Fn rearrangement. The principle of the Matlab analysis of the Fn rearrangement is explained in this figure. A) Firstly, the fluorescence intensity of a Fn coated surface without cells was measured. The histogram gave the intensity values of the Fn background. The upper and lower intensity values were set as higher and lower intensity thresholds. The intensity of fluorescence on a Fn coated surface with a cell was then measured. B) The area below the lower threshold is colored blue, indicating removed Fn, the area above the upper threshold is red, indicating assembled Fn. The sum of these two values corresponded to the total rearranged surface area.
(0.32 MB JPG)

Table S1 Overview of geometries and dimensions of pattern shapes. To give an indication which surface area corresponds to what size of a certain pattern, we present diameter/side length, projected surface are and total surface area of some selected patterns. Please note that this list is not a complete list of all the patterns used. It would be impossible to present all the exact dimensions in a clearly arranged way since we often varied the side length/radius by only 1 µm. Nevertheless, this list should help you to estimate the pattern size.
(0.06 MB DOC)
