# Supplementary figures and images for: Dimensionality Controls Cytoskeleton Assembly and Metabolism of Fibroblast Cells in Response to Rigidity and Shape
Source: PLoS One. 2010 Mar 23;5(3):e9445. doi: 10.1371/journal.pone.0009445 (PMC2843632; doi:10.1371/journal.pone.0009445)

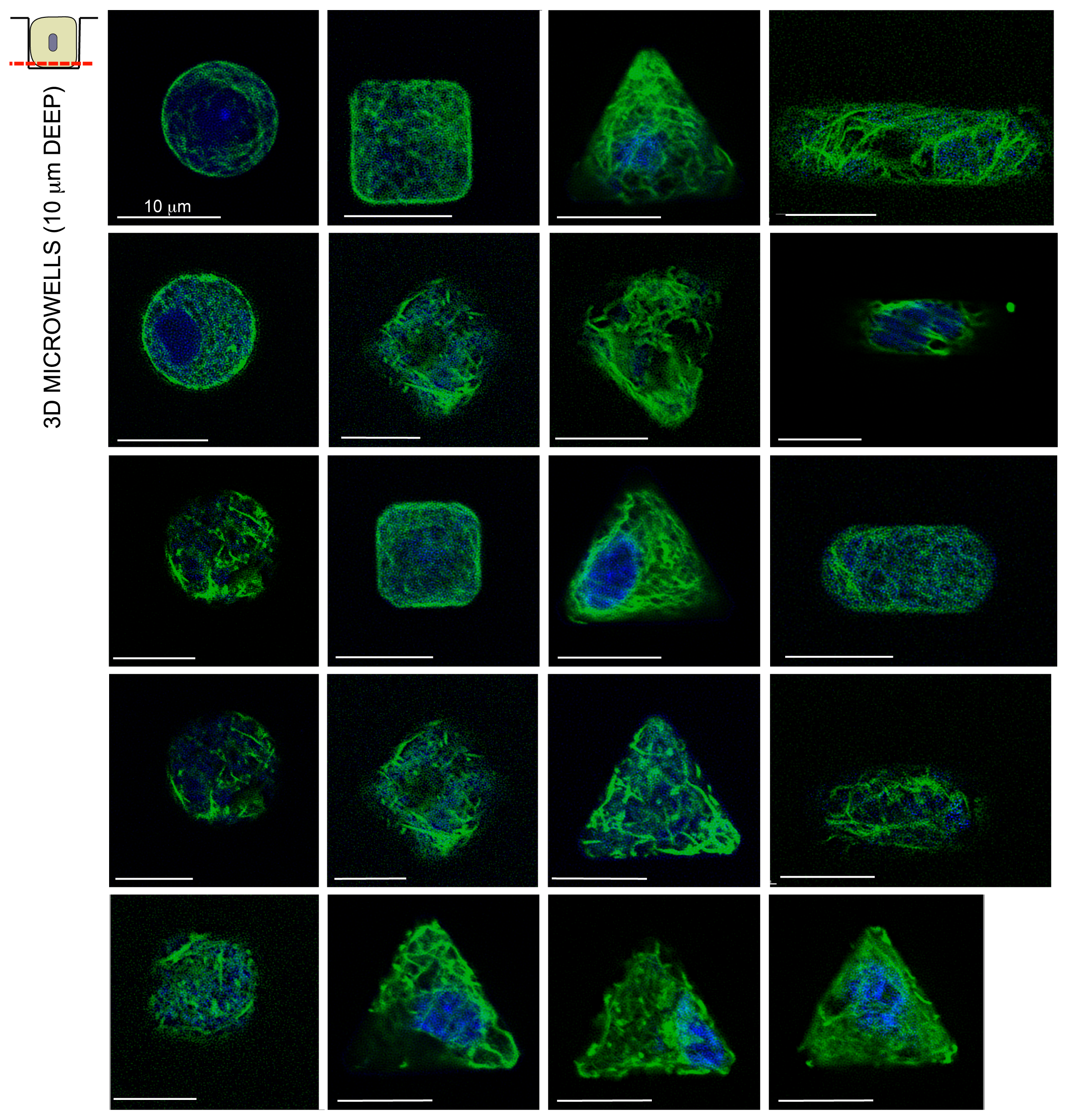

Supplement: Figure S1 — Library of different microwell shapes and actin formation in 10 µm deep, hard wells. Actin filament assembly of primary fibroblast (HFF) cells was visualized using phalloidin Alexa 488 (green), the nucleus with ethidium-homodimer (blue). This figure shows the reproducibility of the actin formation inside small, 10 µm deep microwells. This figure provides a library of different cells inside microwells (circles, squares, triangles, rectangles). (5.14 MB TIF) [file pone.0009445.s001.tif]

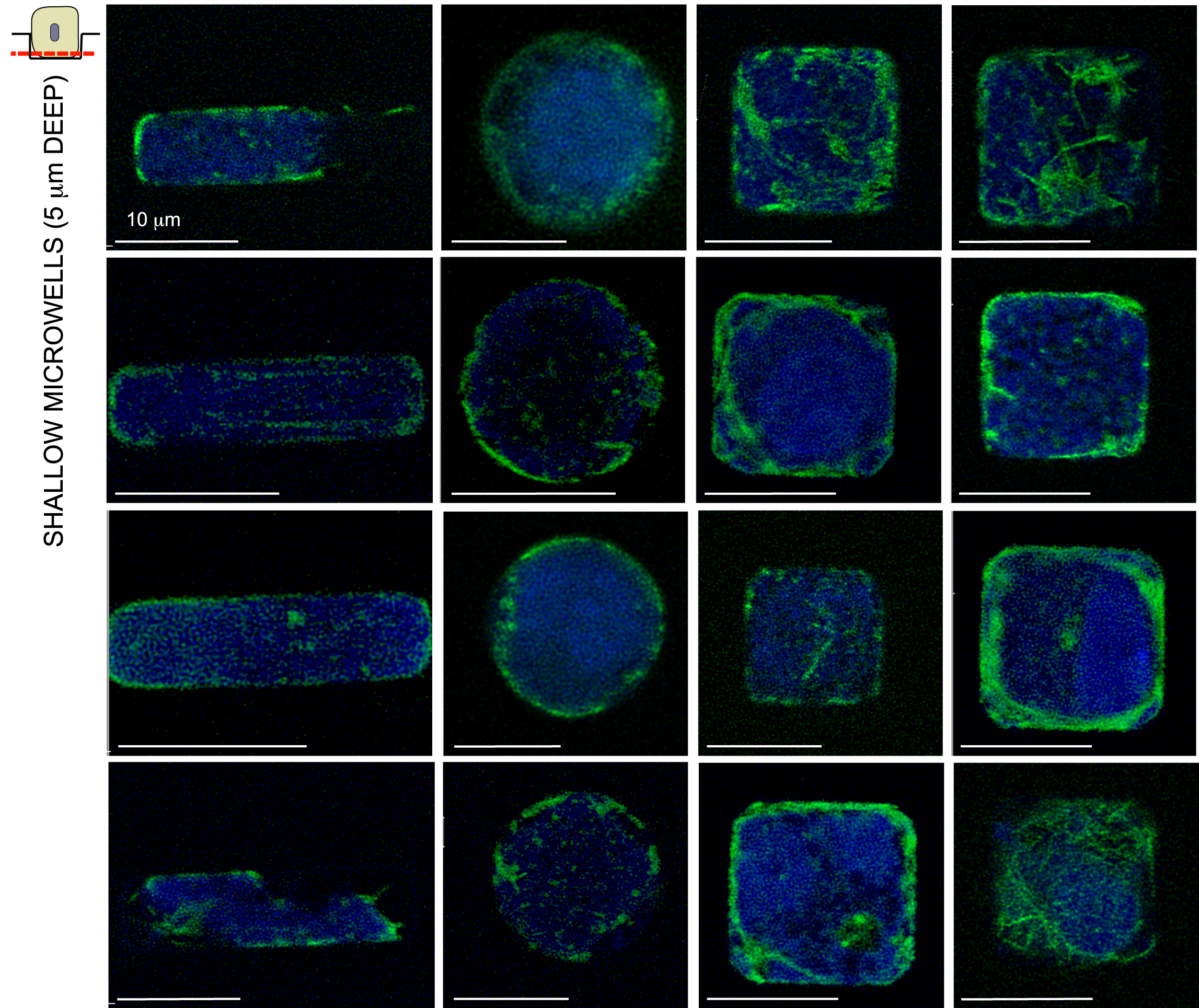

Supplement: Figure S2 — Library of actin skeleton of cells inside shallow microwells (5 µm). Actin filament assembly of primary fibroblast (HFF) single cells was visualized using phalloidin Alexa 488 (green), the nucleus with ethidium-homodimer (blue). It shows the actin formation inside small, 5 µm deep microwells. This figure provides a library of cells inside microwells of different shapes (rectangle, circles, squares). (4.62 MB TIF) [file pone.0009445.s002.tif]

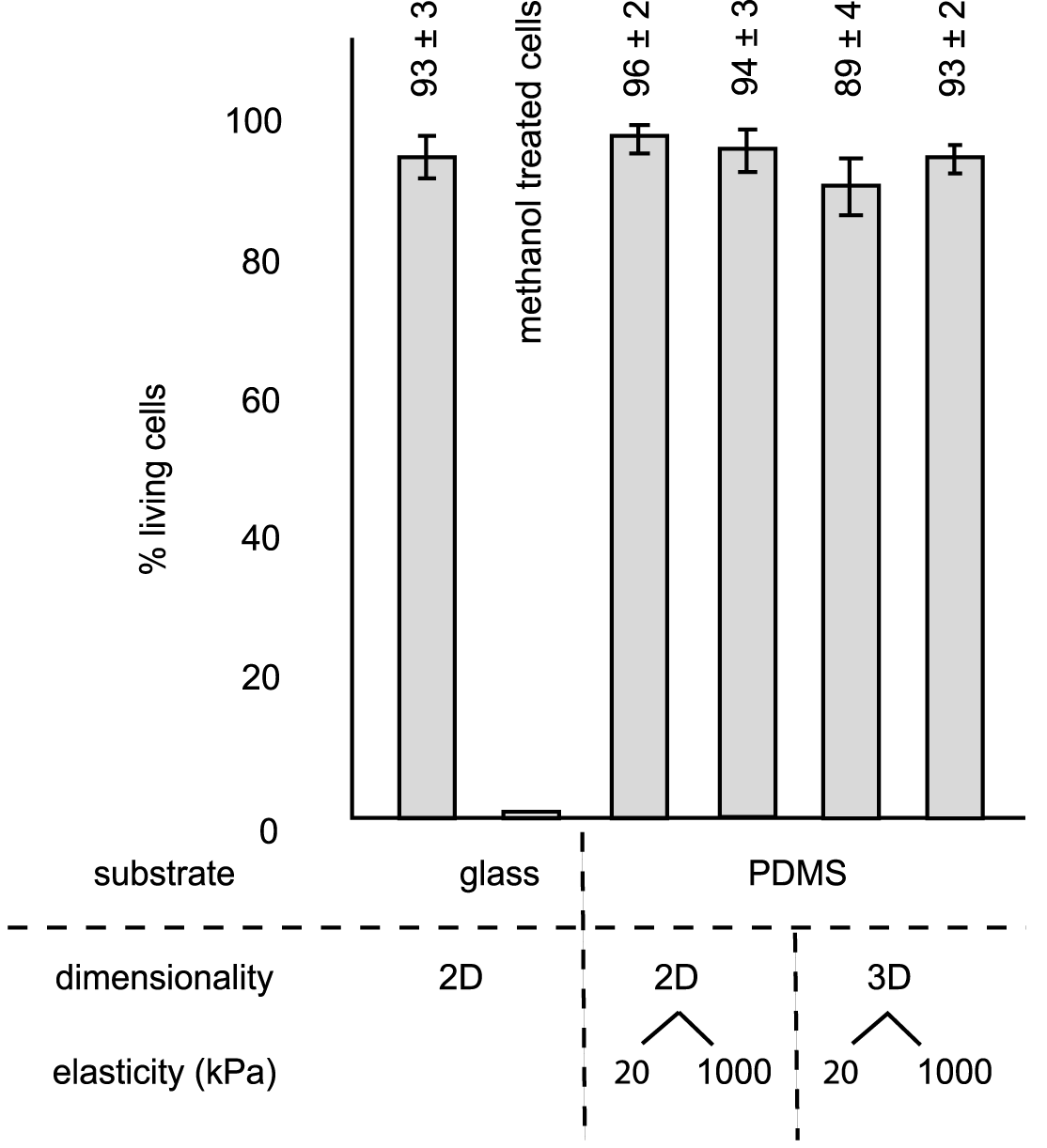

Supplement: Figure S3 — Cell survival of HUVECs in dependence of dimensionality and PDMS stiffness. Human umbilical vein endothelial cells (HUVECs) were cultivated on 2-D and inside 3-D microwells on both soft (20 kPa) and hard (1 MPa) PDMS. This shows that the stiffness of the PDMS does not reduce the cell survival. (0.14 MB TIF) [file pone.0009445.s003.tif]

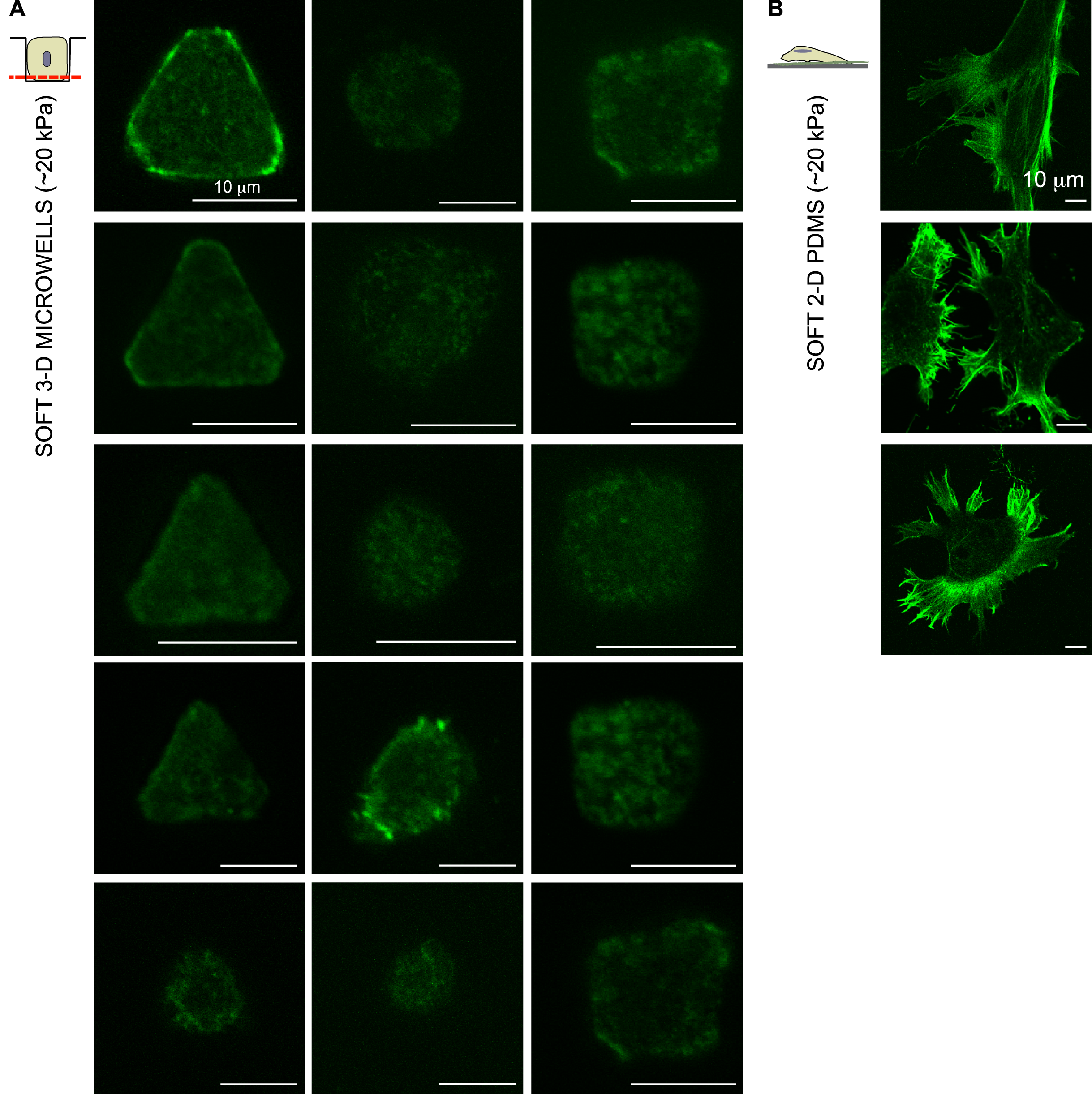

Supplement: Figure S4 — Library of actin skeleton of cells inside 10 µm deep, soft (20 kPa Young's Modulus) microwells. Actin filament assembly of primary fibroblast (HFF) cells was visualized using phalloidin Alexa 488 (green). The figure shows the reproducibility of the A) hindrance of actin filament assembly inside soft, 10 µm deep (∼20 kPa) microwells. This figure provides a library of different cells inside microwells (circles, squares, triangles). B) Cells on a flat 2-D, homogeneously coated substrate of the same stiffness showed clear actin filament formation. (3.24 MB TIF) [file pone.0009445.s004.tif]

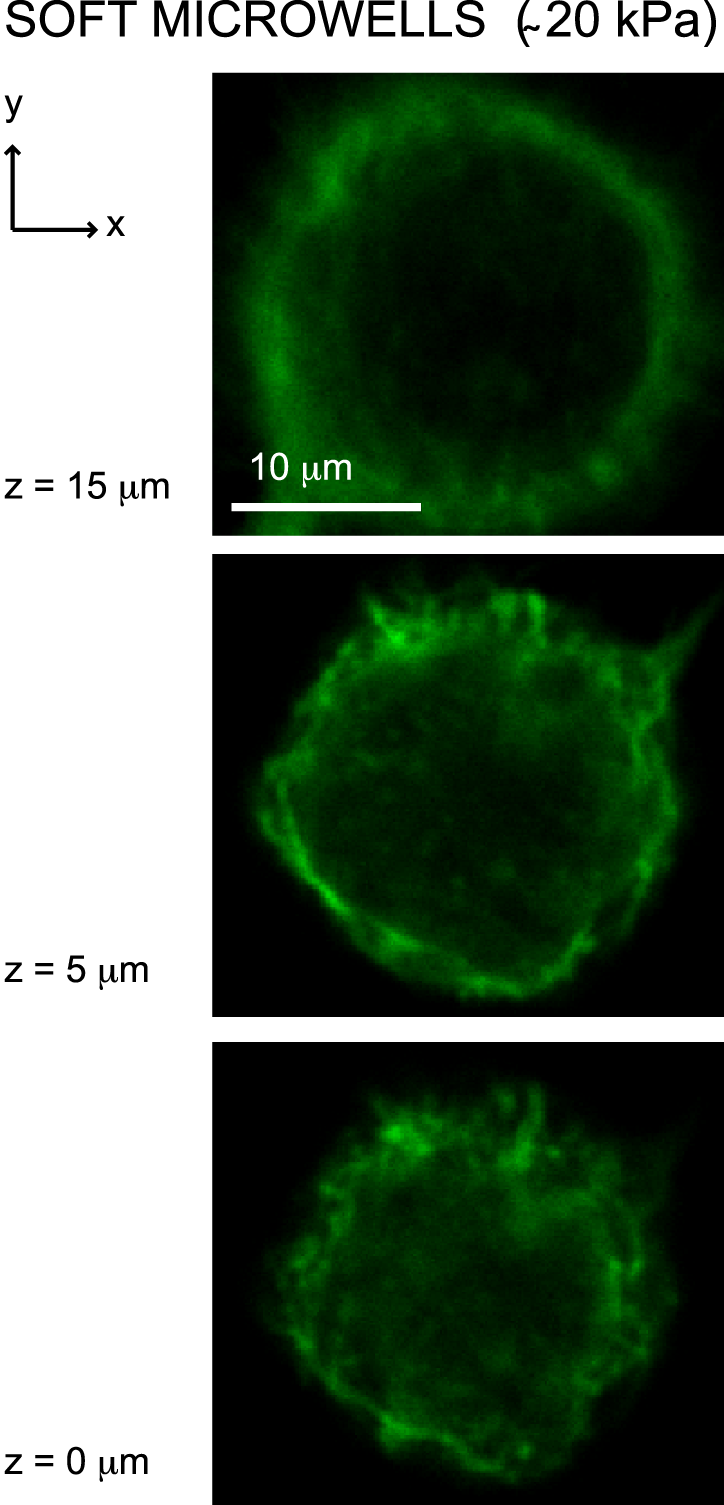

Supplement: Figure S5 — Stack of actin skeleton of cells inside a soft microwells (20 kPa). Actin filament assembly of primary fibroblast (HFF) single cells was visualized using phalloidin Alexa 488 (green). It shows the absense of actin fibers inside a soft (20 kPa) microwell in contrast to hard microwells. This figure demonstrates the lack of actin fibers on the different levels inside the cells. (0.42 MB TIF) [file pone.0009445.s005.tif]

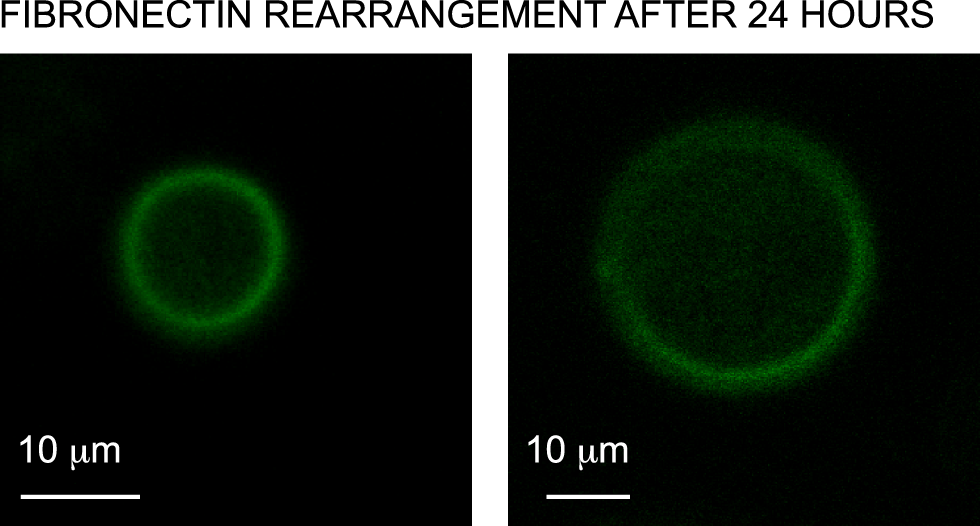

Supplement: Figure S6 — Rearrangement of surface adsorbed Fn in 3-D after 24 hours. The influence of time on surface adsorbed Fn rearrangement (Fn, fluorescently labeled with Alexa 488) was investigated on 3-D microwell culture substrates after 24 hours in cell culture. After 24 there was no rearrangement of surface adsorbed Fn observable which corresponded to substrates after 90 minutes. (0.29 MB TIF) [file pone.0009445.s006.tif]

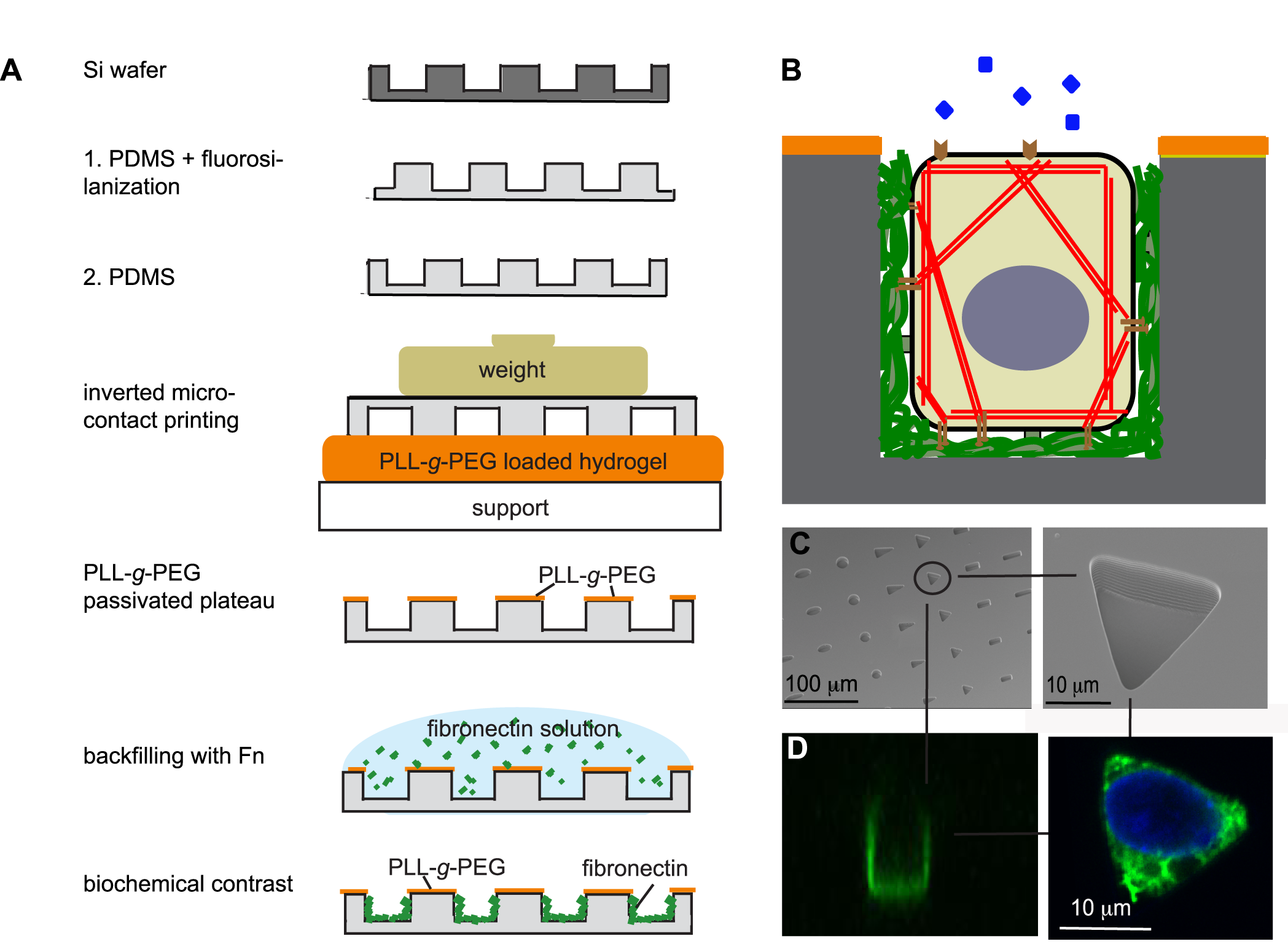

Supplement: Figure S7 — Production by replication and surface modification of the microwell substrates. A) First, the microwell structure of the silicon wafer (positive structure) was replicated into PDMS with the latter serving as master (negative structure) for the subsequent replication step. After fluorosilanization of the PDMS master, the microwell structure was replicated into a thin PDMS supported on a glass cover slip (total thickness<170 µm, compatible with inverted-stage high-resolution fluorescence microscopy). The microwell plateau surface was next passivated by inverted micro-contact printing of PLL-g-PEG using a polyacrylamide stamp. The stamp was placed upside down on the structured surface, and a 5 g weight was applied. After passivation of the plateau, the sample was finally exposed to a solution containing fibronectin (Fn) for coating of the wells; after rinsing only the microwell surfaces (bottom and walls) were coated with the cell-adhesive protein. B) Cartoon of microwells with well volumes that can be tailored to the volume of a single cell resulting in single cells in wells that interact with ECM molecule inside the well while the plateau areas resist protein adsorption and cell adhesion. C) Scanning electron microscopy images of the microwell surface show wells with different shapes and a zoom into a single triangular well. D) The scanning laser confocal microscope image shows the side view of a microwell selectively coated with fluorescently labeled Fn (green). E) A fibroblast cell adhering inside a microwell was visualized with actin (Phalloidin 488, green) and nuclei staining (ethidium homodimer, blue). (0.70 MB TIF) [file pone.0009445.s007.tif]

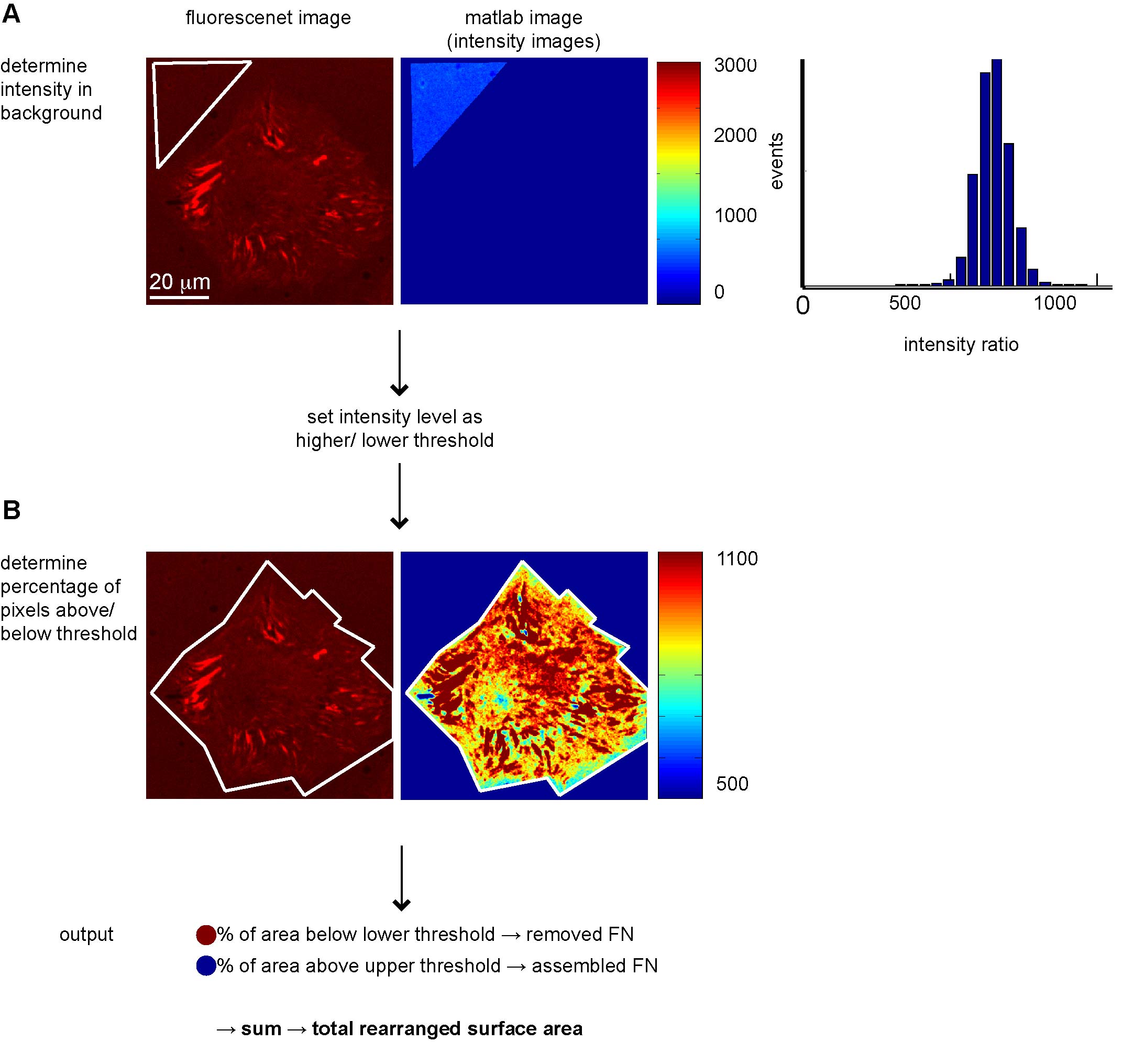

Supplement: Figure S8 — Analysis of Fn rearrangement. The principle of the Matlab analysis of the Fn rearrangement is explained in this figure. A) Firstly, the fluorescence intensity of a Fn coated surface without cells was measured. The histogram gave the intensity values of the Fn background. The upper and lower intensity values were set as higher and lower intensity thresholds. The intensity of fluorescence on a Fn coated surface with a cell was then measured. B) The area below the lower threshold is colored blue, indicating removed Fn, the area above the upper threshold is red, indicating assembled Fn. The sum of these two values corresponded to the total rearranged surface area. (0.32 MB JPG) [file pone.0009445.s008.jpg]
