# Supplementary material for: Dimensionality Controls Cytoskeleton Assembly and Metabolism of Fibroblast Cells in Response to Rigidity and Shape
Source: PLoS One. 2010 Mar 23;5(3):e9445. doi: 10.1371/journal.pone.0009445 (PMC2843632; doi:10.1371/journal.pone.0009445)
Supplement: Table S1 — Overview of geometries and dimensions of pattern shapes. To give an indication which surface area corresponds to what size of a certain pattern, we present diameter/side length, projected surface are and total surface area of some selected patterns. Please note that this list is not a complete list of all the patterns used. It would be impossible to present all the exact dimensions in a clearly arranged way since we often varied the side length/radius by only 1 µm. Nevertheless, this list should help you to estimate the pattern size. (0.06 MB DOC) [file pone.0009445.s009.doc]

**Tab. S1 Overview of geometries and dimensions of pattern shapes.** To give an indication which surface area corresponds to what size of a certain pattern, we present diameter/ side length, projected surface are and total surface area of some selected patterns. Please note that this list is not a complete list of all the patterns used. It would be impossible to present all the exact dimensions in a clearly arranged way since we often varied the side length/ radius by only 1 μm. Nevertheless, this list should help you to estimate the pattern size.

| **shape** | **side length/ diameter (μm)** | **projected surface area (μm^2^)** | **total surface area (μm^2^)** |
| --- | --- | --- | --- |
| **circle** | 10 | 78 | 392 |
|  | 20 | 314 | 942 |
|  | 25 | 490 | 1275 |
|  |  |  |  |
| **square** | 10 | 100 | 500 |
|  | 15 | 225 | 825 |
|  | 20 | 400 | 1200 |
|  | 30 | 900 | 2100 |
|  |  |  |  |
| **triangle** | 15 | 97 | 547 |
|  | 20 | 173 | 773 |
|  | 25 | 270 | 1020 |
|  |  |  |  |
| **rectangle** | 8 x 16 | 128 | 608 |
|  | 10 x 20 | 200 | 800 |
|  | 15 x 30 | 450 | 1350 |
